# Structure and function of the Orc1 BAH-nucleosome complex

**DOI:** 10.1038/s41467-019-10609-y

**Published:** 2019-07-01

**Authors:** Pablo De Ioannes, Victor A. Leon, Zheng Kuang, Miao Wang, Jef D. Boeke, Andreas Hochwagen, Karim-Jean Armache

**Affiliations:** 1 Skirball Institute of Biomolecular Medicine, Department of Biochemistry and Molecular Pharmacology, New York University School of Medicine, New York, NY 10016 USA; 2 Department of Biology, New York University, New York, NY 10003 USA; 3 Institute for Systems Genetics, Department of Biochemistry and Molecular Pharmacology, NYU Langone Health, New York, NY 10016 USA; 4 Present Address: Department of Immunology, University of Texas Southwestern Medical Center, Dallas, TX 75390 USA

**Keywords:** X-ray crystallography, Chromatin structure, Histone post-translational modifications, Nucleosomes, Double-strand DNA breaks

## Abstract

The Origin Recognition Complex (ORC) is essential for replication, heterochromatin formation, telomere maintenance and genome stability in eukaryotes. Here we present the structure of the yeast Orc1 BAH domain bound to the nucleosome core particle. Our data reveal that Orc1, unlike its close homolog Sir3 involved in gene silencing, does not appear to discriminate between acetylated and non-acetylated lysine 16, modification states of the histone H4 tail that specify open and closed chromatin respectively. We elucidate the mechanism for this unique feature of Orc1 and hypothesize that its ability to interact with nucleosomes regardless of K16 modification state enables it to perform critical functions in both hetero- and euchromatin. We also show that direct interactions with nucleosomes are essential for Orc1 to maintain the integrity of rDNA borders during meiosis, a process distinct and independent from its known roles in silencing and replication.

## Abstract (web summary)

The Origin Recognition Complex (ORC) plays conserved and diverse roles in eukaryotes. Here the authors present the structure of a chromatin interacting domain of yeast Orc1 in complex with the nucleosome core particle, revealing that Orc1 interacts with the histone H4 tail irrespective of K16 acetylation; a modification that regulates accessibility to chromatin.

## Introduction

The six-subunit origin recognition complex (ORC) plays diverse and conserved roles in eukaryotic genomes, such as replication initiation and recruitment of silencing proteins to establish heterochromatin^1–7^. Recently, it was found that the ORC subunit Orc1 protects rDNA borders from double-strand breaks during meiosis^8^. All these seemingly diverse functions entail ORC interactions with chromatin. In this context, Orc1 is of particular interest as it contains a bromo-adjacent homology (BAH) domain, a nucleosome interacting module, at its N-terminus^9,10^.

Genome-wide studies in *Saccharomyces cerevisiae* revealed that the Orc1 BAH domain contributes to ORC association with most yeast origins of replication and characterized a class of origins that require this domain for normal activity^11^. The role of this domain is also well-defined at yeast heterochromatin-like domains which include those found at silent mating-type loci *HML* and *HMR*, and at telomeres. At *HM* loci, the Orc1 BAH domain binds at the DNA silencer element and recruits the adapter protein Sir1, which establishes silencing by recruiting the silent information regulator (SIR) complex that contains Sir2, Sir3, and Sir4 proteins^12–18^. Mutations and deletions of the Orc1 BAH domain that disrupt Sir1 interactions result in epigenetic bistability of this locus^19^. At telomeres, the BAH domain is also important for silencing and while the mechanism is less clear, similarly to *HM* loci it depends on N-terminal acetylation of Orc1 by NatA-Ard acetyltransferase^20^.

Budding yeast Orc1 BAH domain most closely resembles the BAH domain of Sir3 (48% identity and 67% similarity), which is not surprising given that these two proteins originated via gene duplication^21,22^. The Sir3 BAH domain plays a critical role in epigenetic silencing in *S*. *cerevisiae*^14,23–26^. It binds to nucleosomes and stabilizes interactions of the SIR complex with chromatin^25,27^. Covalent modifications of histones, specifically acetylation of lysine 16 of histone H4 (H4K16) by Sas2 and deacetylation by Sir2 are critical determinants of the silenced state^28–30^. The structure of the Sir3 BAH domain in complex with the nucleosome revealed the role of these modifications at an atomic level^31–34^. While genetic, biochemical and structural analyses provided in depth description of Sir3 interactions with nucleosomes, it is much less clear how ORC binds to nucleosomes, and how its binding is regulated by histone modifications.

In higher eukaryotes, ORC interacts with a variety of histone modifications absent from budding yeast, including H3K27me3, H3K9me3, and H4K20me2^35–37^. For proper function, Orc1 as part of the ORC complex must bind at two dramatically different types of chromatin: closed and inaccessible regions within heterochromatin as well as open and accessible regions within euchromatin. In all eukaryotes including budding yeast, H4K16 acetylation status determines the conformation and accessibility of chromatin structure^38,39^; it is not clear, however, how Orc1/chromatin interactions are regulated by this modification. While it has been observed that sensitivity toward H4K16 modification status differs between Orc1 and Sir3^25^, the mechanisms are not known. In addition, while it is clear that ORC binds within chromatin, if and at which loci direct nucleosome interactions are required for its function is not well established. One example of such ambiguity corresponds to the protection of rDNA from genomic rearrangements. The borders of the rDNA are particularly susceptible to meiotic double-strand breaks (DSBs) and nonallelic homologous recombination (NAHR). In budding yeast, Orc1 is involved in protecting these regions, and deletion of its BAH domain leads to increased rates of DSBs and NAHR at this locus, resulting in a perturbed number of rDNA repeats in progeny^8^. It is not known whether this function depends on direct interactions between the BAH domain and nucleosomes or on recruitment of another set of proteins via this domain.

Given the functional conservation of the ORC complex in eukaryotes, the general conservation of mechanisms that govern chromatin conformation, and multiple diseases that are associated with perturbed ORC activity, it is essential to understand the interactions between Orc1 BAH and nucleosomes and their regulation by posttranslational histone modifications.

We determined the X-ray structure of *S*. *cerevisiae* Orc1 BAH domain in complex with a nucleosome at 3.3 Å resolution. The structure revealed altered interactions with the histone H4 N-terminal tail when compared with Sir3 and suggested that Orc1 does not discriminate between acetylation states of H4 lysine 16. Comprehensive biochemical characterization confirmed and helped to elucidate the mechanism for this unique feature of Orc1. We discovered that direct interactions of Orc1 with nucleosomes are essential to maintaining the integrity of rDNA borders during meiosis. In support of this finding, we show that mutations that lower the affinity of the Orc1-BAH domain for nucleosomes results in accumulation of double-strand breaks at this locus. We also show that this newly discovered role of Orc1-nucleosome interactions is distinct and independent from its known roles in silencing and replication. Together this study suggests a mechanism that allows Orc1 to bind nucleosomes across the genome irrespective of the acetylation status of histone H4 and reveals a process where direct interactions between Orc1 BAH domain and nucleosomes are critical.

## Results

### Structure determination of the Orc1 BAH-nucleosome complex

**Fig. 1:**
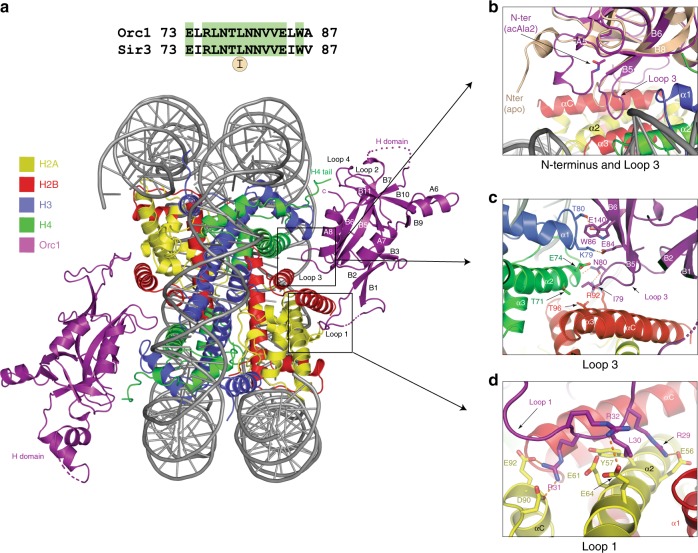
Overview of the structure and main interfaces. **a** Sequence alignment of Loop 3 in Orc1 and Sir3 with conserved leucine 79 (Top). Front view of the Orc1 BAH-Nucleosome complex (Bottom). The structure is color coded (BAH in purple, H2A in yellow, H2B in red, H3 in blue, H4 in green, and DNA in gray). **b**–**d** Main interfaces between Orc1 BAH and nucleosome are indicated in squares. **b** Conformation of acetylated N-terminus of Orc1. Superposition of the unacetylated, apo Orc1 structure (from PDB ID: 1M4Z; light brown) shows structural rearrangements of the N-terminus of the BAH domain. **c** Orc1 interactions with LRS region on nucleosome. **d** Details of Loop 1 interactions with acidic patch on the nucleosome. Important residues are highlighted and depicted in sticks. Hydrogen bonds are shown as dashed lines

To understand how Orc1 interacts with chromatin, we carried out structural studies of the *S. cerevisiae* Orc1 BAH domain in complex with the nucleosome. After the initial trials using bacterially or insect cell expressed wild-type protein failed, we turned to the L79I mutant. This dominant mutation was found in Sir3 in a screen focused on suppressors of the loss of telomeric and rDNA silencing phenotype caused by the histone H3A75V mutation, suggesting an increased affinity for nucleosomes^40^. As this residue is conserved between Orc1 and Sir3 (Fig. 1a, Top), we expressed the Orc1 BAH L79I mutant in insect cells for our studies. We report the crystal structure of the complex of the Orc1 BAH L79I mutant in complex with the nucleosome core particle determined at 3.3 Å resolution.

### General architecture of the complex

The structure shows the BAH domain bound at each of the two opposite faces of the nucleosome^41^, creating a pseudo-two-fold symmetry (Fig. 1a, Bottom), similar to that seen with the Sir3-nucleosome complex (Supplementary Fig. 1)^31–34^. The Orc1 BAH domain forms extensive interactions with histones H2A, H2B, H3, and H4. The only observed contacts with DNA are that with the symmetry related complex. Interactions with the core histones are mediated through several distinct regions on the surface of the BAH domain, burying a surface of 1590 Å^2^, an interface which is slightly smaller than that seen in Sir3-nucleosome complex structures^31^. The Orc1 BAH domain interacts with the H4 tail, the LRS (Loss of rDNA Silencing) domain composed of histones H3 and H4, with the region of H2B adjacent to the LRS surface as well as the H2A/H2B acidic patch (Fig. 1a and Supplementary Fig. 2)^42^. Several regions show folding transitions upon complex assembly, both in the BAH domain and in the nucleosome. In total, 22 residues of the BAH domain make direct contacts with core histones using a cut off distance of 4.1 Å (Supplementary Fig. 2). The smaller number of interacting residues and consequently a decrease in buried surface compared with Sir3 structures are in part due to differences in interactions between the BAH domain and the H4 tail, discussed below. Complex A and monomer K were used hereafter for structure analysis, due to better ordering compared with the rest of the chains.

### The interface between Orc1 and the nucleosome

In yeast, acetylation of the N-terminus (N^α^-acetylation) of Orc1 by the NatA-Ard complex regulates its chromatin role^20,43^. Here we show that N^α^-acetylation plays a direct role in nucleosome binding. The acetylated N-terminus is visible in each of the ordered BAH domains and displays a large conformational change when compared with the unacetylated apo-Orc1 structure (Fig.1b). Specifically, Cα atoms of Loop 3 show a maximum movement of 9 Å toward the nucleosome to adopt an optimal configuration for interaction (Fig. 1b). The acetyl group projects into a hydrophobic pocket formed between strand B6 and the loop connecting strand B8 and helix A5, stabilizing the conformation of Loop 3. A similar N-terminal configuration has been observed in acetylated Sir3 BAH structures, indicating a conserved mechanism that regulates association of these domains with chromatin^32,44^. One of the largest interfaces between Orc1 and the nucleosome is formed by contacts with the LRS surface of histones H3 and H4, and H2B (Fig. 1c). Here, Orc1 W86 is involved in hydrophobic interactions with the backbone of several H3 residues including Q76, D77, F78, K79, and T80 (Fig.1c). In addition, the Orc1 R75 side chain makes a hydrogen bond with the main chain of H3 D77, while E84 and E140 of Orc1 form hydrogen bonds with H3 residues K79 and T80, respectively. In the same interface, Orc1 Loop 3 residues I79 and N80 establish interactions with residues in histone H4 and H2B, respectively. Particularly, the side chain of I79 makes VdW interactions with a hydrophobic cavity formed by side chains of H4 T71 as well as H2B T96 (Fig. 1c). This residue, L79 in the wild-type protein, was mutated to isoleucine to increase the affinity of Orc1 to nucleosome. Orc1 Loop 3 shows a 1.0 Å Cα movement toward the surface of the nucleosome when compared with the Sir3-nucleosome structure. In addition, the Orc1 N80 side chain makes a potential hydrogen bond with the backbone of H4 E74, which is at the end of Helix α2, and a hydrogen bond with the side chain of H2B R92 (Fig. 1c). Moving down in this interface, Loop 1 interacts via charge complementarity with the nucleosome acidic patch (Fig. 1d). Specifically, Orc1 residues R29, R31 and R32 establish a network of salt bridges with acidic residues E56, E61, E64, D90, and E92 from H2A (Fig. 1d). This region emerged as a hot spot for interactions between various chromatin associated proteins and nucleosomes^31,45–47^.

### Recognition of the histone H4 tail and K16 by Orc1

**Fig. 2:**
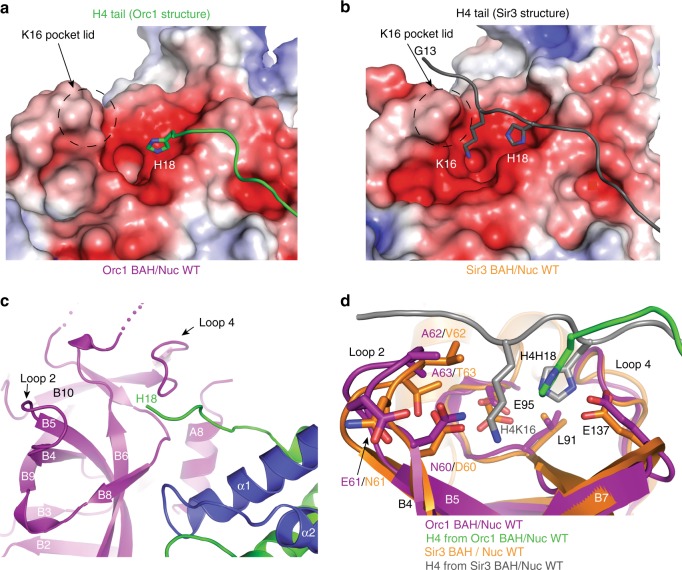
H4 tail recognition by Orc1 and Sir3 BAH domains. **a** Electrostatic surface potential representation of Orc1 in complex with the nucleosome (positive, blue; negative, red). Histone H4 tail, green. **b** Electrostatic surface potential representation of Sir3 BAH in complex with the nucleosome (PDB ID: 4KUD). Histone H4 tail, gray. **c** Overview of H4 tail interface highlighting the secondary structure elements in histones and in Orc1. **d** Detailed view of superimposed pockets of Orc1 BAH domain (purple) and Sir3 BAH domain (orange) from the respective nucleosome complex structures. H4 tail in Orc1 structure (green) and in Sir3 structure (gray). Residues that make important contacts are depicted as sticks

The histone H4 tail has well-established roles in chromatin condensation^29,31,33,39,48^. In the Orc1 BAH-nucleosome structure, part of the histone H4 tail is ordered and visible in the electron density map (Fig. 2a and Supplementary Fig. 3). H4 interacts with the BAH domain of Orc1 via charge complementarity as observed in the Sir3 BAH domain structure. The negatively charged BAH domain presents a favorable surface for positively charged residues on histone H4 (Fig. 2a, b). The main interface in this region is created by Loop 4 that binds the nucleosome proximal portion of the histone H4 tail (Fig. 2c). This part of the histone H4 tail binds in a similar manner in both Sir3 and Orc1 (Supplementary Fig. 4) helping anchor the tail to the BAH domain surface. The most striking feature of the Orc1-nucleosome structure is the absence of an ordered H4K16 side chain in the electron density map of all copies of Orc1 in the asymmetric unit. The first residue of histone H4 visible in the electron density is H18 (Fig. 2a and Supplementary Fig. 3), which binds in the pocket on the surface of the Orc1 BAH domain. We were puzzled by this finding since Sir3 has a well-ordered lysine 16 of histone H4 in this pocket, and the tail extends to residue G13 (Fig. 2b and Supplementary Fig. 4). In general, the pocket is relatively well conserved in both structures. H4H18 forms contacts with Orc1 E95, E137, and L91 (Fig. 2d). What is the reason then for the lack of density for K16 in Orc1 structure? The pocket appears more open in Orc1 when compared with Sir3, where Loop 2 forms a Lid that caps K16 (Fig. 2a, b). In addition, we identified a stretch of residues in the Lid (residues 60–63) that are not conserved between Orc1 and Sir3 (Supplementary Fig. 2). The residues in Sir3 that could form VdW interactions with the aliphatic moiety of K16 are substituted by smaller residues in Orc1, which consequently made the pocket appear more open (Fig. 2d). Notably, mutation of the Lid residue T63 in Sir3 to alanine (Orc1-like mutation) results in dominant loss of silencing perhaps due to perturbed interactions of Sir3 with unacetylated K16 in this mutant^49^.

**Fig. 3:**
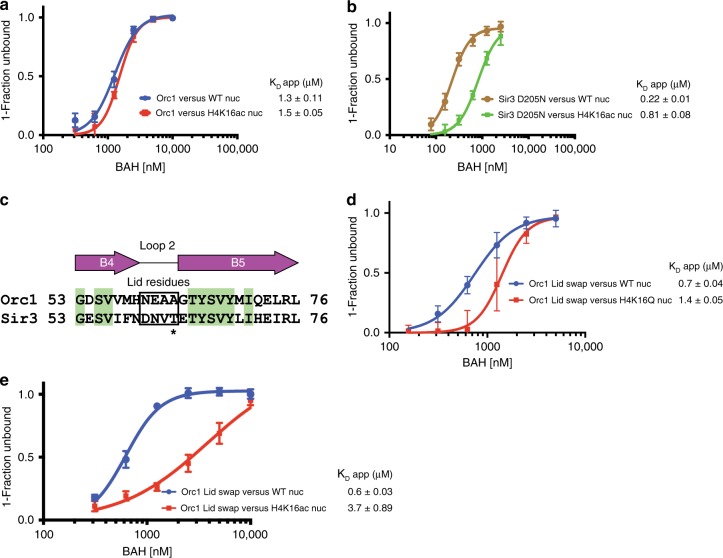
Orc1 does not discriminate the acetylation state of H4K16. **a** Binding of Orc1 BAH domain (Orc1) to unmodified (WT nuc) and H4K16ac nucleosomes measured by EMSA. **b** Binding of Sir3 D205N BAH domain to unmodified and H4K16ac nucleosomes measured by EMSA. **c** Sequence alignment of variable Loop 2 region in Orc1 and Sir3. Loop 2 Lid residues are highlighted in a box. Asterisk represents a dominant loss of silencing mutation in Sir3. **d** Binding of Orc1 with Sir3-swapped residues in the Lid of Loop 2 to unmodified or H4K16Q (acetyl mimic) nucleosomes measured by EMSA. **e** Binding of Orc1 with Sir3-swapped residues in the Lid of Loop 2 to unmodified and H4K16ac nucleosomes measured by EMSA shows that these residues confer discrimination for H4K16. Each data point and error bar represent the mean ± s.d. from three or more independent experiments. The standard errors of dissociation constants (*K*_D_) are indicated

Based on the structure and previous observations^25^, we hypothesized that Orc1 is less sensitive toward the modification status of K16 than Sir3. To address whether this is true in solution, we performed binding measurements of Orc1 with wild-type and H4K16ac nucleosomes^50,51^ using EMSAs. We found that Orc1 displays similar affinity for both wild-type and H4K16ac nucleosome (*K*_D_ app 1.3 μM and 1.5 μM, respectively) (Fig. 3a and Supplementary Fig. 6). While it is known that Sir3 shows a strong preference for unmodified K16, we wanted to confirm that under our experimental conditions. As the Sir3 BAH domain (construct 1–214) under our EMSA conditions does not form a stable complex with the nucleosome, we used a Sir3 BAH D205N hypermorphic mutant which binds to nucleosomes with higher affinity^27,44^. We found that Sir3 D205N displays a clear preference for unmodified nucleosomes (WT nuc) (3.7-fold increase) relative to affinity for the H4K16ac nucleosomes (Fig. 3b and Supplementary Fig. 6). We also corroborated these findings using H4K16Q (acetyl mimic) nucleosomes (Supplementary Fig. 6). Therefore, our in vitro studies strongly suggest that the Orc1 BAH domain does not discriminate between acetylated and unacetylated H4K16. To address whether this is a consequence of amino acid changes in the Loop 2 Lid, we generated a Lid swap mutant. In this mutant, Orc1 residues 60–63 (NEAA) were replaced with the corresponding residues in Sir3 (DNVT) (Fig. 3c). Then, we tested this mutant’s ability to discriminate between modification state of H4K16 nucleosomes by EMSA (Fig. 3d, e and Supplementary Fig. 6). First, we tested the mutant’s ability to discriminate between wild-type and H4K16Q (acetyl mimic) nucleosomes (Fig. 3d and Supplementary Fig. 6). Binding experiments showed that the mutant binds unmodified nucleosomes with a small but reproducible increase in affinity (1.9-fold increase when compared with Orc1 WT binding to unmodified nucleosomes, Fig. 3a), while the affinity of the same mutant for H4K16Q nucleosomes was markedly decreased (2.0-fold decrease when compared with binding to unmodified nucleosome, Fig. 3d). Moreover, the effect of the modification was more pronounced when we measured the affinity for the more physiological substrate: H4K16ac nucleosomes (Fig. 3e), which was decreased (2.6-fold decrease when compared with Orc1 swap mutant binding to H4K16Q nucleosome, Fig. 3d). This resulted in roughly sixfold preference for unmodified nucleosomes when compared with K16 acetylated substrate for this mutant (Fig. 3e). These results corroborate our hypothesis that residues present in the Sir3 Lid present stronger contact with the N-terminal portion of the H4 histone tail, specifically with K16, than the corresponding residues in Orc1. The discrimination against acetylated K16 by Sir3 could result from electrostatic and steric effects. The more open pocket as well as its inability to form stabilizing VdW interactions would render Orc1 unable to discriminate the acetylation states of this residue.

To evaluate the acetylation state of nucleosomes surrounding Orc1 binding sites at origins of replication (*S. cerevisiae* ARS sequences), we reanalyzed ChIP-seq data from prior studies^52,53^. First, we aligned ARS consensus sequences (ACS) of all yeast ARSs with Orc1 binding signals by aligning the first nucleotide of the ACS in the center and orienting T-rich strands on the top (forward) strand^54,55^. Then, we evaluated prior datasets of total histone H3 to assess the overall nucleosome density and H4K16ac to evaluate acetylation state. These analyses confirmed that Orc1 binding sites are generally nucleosome-depleted (Supplementary Fig. 5). However, they also revealed that the origins in silent chromatin (telomeres and *HM* loci) show very low levels of H4K16 acetylation, presumably as the consequence of ongoing deacetylation by the abundance of Sir2 found in those regions (Supplementary Fig. 5), whereas the vast majority of (euchromatic) origins show peaks of acetylated H4K16 at the nucleosome surrounding the sites of Orc1 binding (Supplementary Fig. 5).

Next, we addressed the location of Orc1 with respect to ARS, and as expected Orc1 presents high occupancy at the center of the ARS position (Supplementary Fig. 5). At the silent chromatin, Orc1 is broadly distributed along the 1000 bp ARS window (Supplementary Fig. 5). However, at the non-silent chromatin origins, Orc1 occupancy shows a narrow distribution that suggests interaction with the −1 and +1 ARS flanking nucleosomes, which are to some extent acetylated (Supplementary Fig. 5). Thus, it appears that Orc1’s BAH domain has both the opportunity and the ability to bind nucleosomes in these two states of histone H4 N-termini.

### Balancing the interactions of Orc1 BAH with the nucleosome

**Fig. 4:**
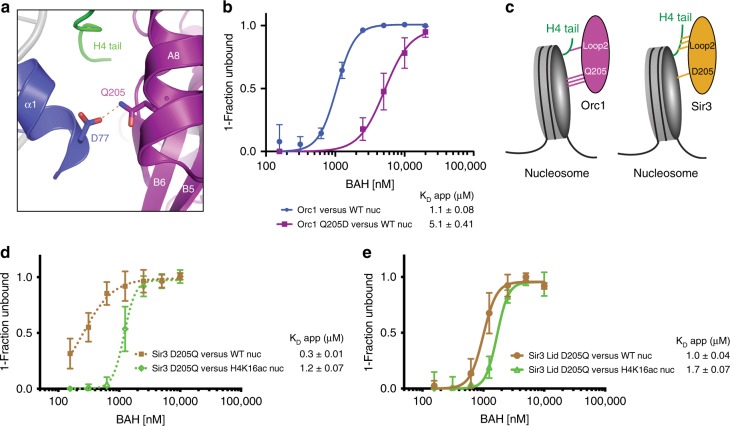
Q205 in Orc1 compensates for the loss of H4 tail interactions. **a** Interaction between Orc1 Q205 (purple) and H3 D77 (blue). **b** Effect of Q205D mutation on Orc1 BAH binding to unmodified nucleosome (WT nuc) as measured by EMSA. **c** Model for Orc1 BAH or Sir3 BAH interactions with nucleosome. Interaction strength is represented by dashes. **d** Binding of Sir3 D205Q to unmodified and H4K16ac nucleosomes measured by EMSA. **e** Binding of Sir3 D205Q with Orc1-swapped residues in the Lid of Loop 2 (Sir3 Lid D205Q) to unmodified and H4K16ac nucleosomes measured by EMSA shows that mutation of these residues reduces the discrimination for H4K16ac. Each data point and error bar represent the mean ± s.d. from three or more independent experiments. The standard errors of dissociation constants (*K*_D_) are indicated

H4 is a critical anchor for Sir3 on the nucleosome^31,56^. Mutations and deletions within the H4 tail as well as K16 modification result in a global loss of interactions between Sir3 and nucleosomes^23,57–59^. Given the fact that Orc1 BAH does not interact with the N-terminal residues of H4, how can it still maintain the overall affinity to maintain productive interactions with nucleosomes? One of the striking differences between Orc1 and Sir3 is found at residue 205. Orc1 has a glutamine at this position (Q205) that is located in Helix A8 and forms a potential hydrogen bond with histone H3 D77 (Fig. 4a). At this position, Sir3 harbors aspartic acid, an acidic residue, mutation of which to a neutral side chain (asparagine) generates the D205N hypermorphic mutation of Sir3^23,27,40,60^. D205N in Sir3 makes the contact with H3 D77 more energetically favorable by removing electrostatic repulsion. Consequently, the D205N mutation significantly increases the affinity of Sir3 for unmodified nucleosomes, a feature critical for structure determination of Sir3 complex with the nucleosome^31^. To determine the importance of this residue in binding of Orc1 BAH to nucleosomes, we mutated glutamine 205 to aspartic acid (Q205D, Sir3-like mutation) and performed EMSA assays with nucleosomes (Fig. 4b and Supplementary Fig. 6). As hypothesized, the Q205D mutation decreased the affinity of Orc1 BAH for nucleosomes suggesting that acquisition of Q at this position balanced the loss of interaction with K16 on histone H4, allowing Orc1 BAH to interact with chromatin (Fig. 4c).

To corroborate the observed differences in nucleosome recognition between Orc1 BAH and Sir3 BAH, we substituted residues in Sir3 with the equivalent residues in Orc1. In binding assays, the D205Q substitution alone makes Sir3 behave similarly to the hypermorphic mutation D205N. Sir3 D205Q binds unmodified nucleosomes with higher affinity than the wild-type domain and discriminates against the H4K16ac modification (4.0-fold difference compared with unmodified nucleosome) (Fig. 4d and Supplementary Fig. 6). As predicted, the replacement of the Lid in the context of D205Q mutation decreases the affinity of Sir3 for the unmodified nucleosome to a level similar to Orc1 WT, and decreases the selectivity for the H4K16ac nucleosome (1.7-fold difference compared with unmodified nucleosome) (Fig. 4e and Supplementary Fig. 6).

### Orc1-nucleosome contacts protect rDNA borders during meiosis

**Fig. 5:**
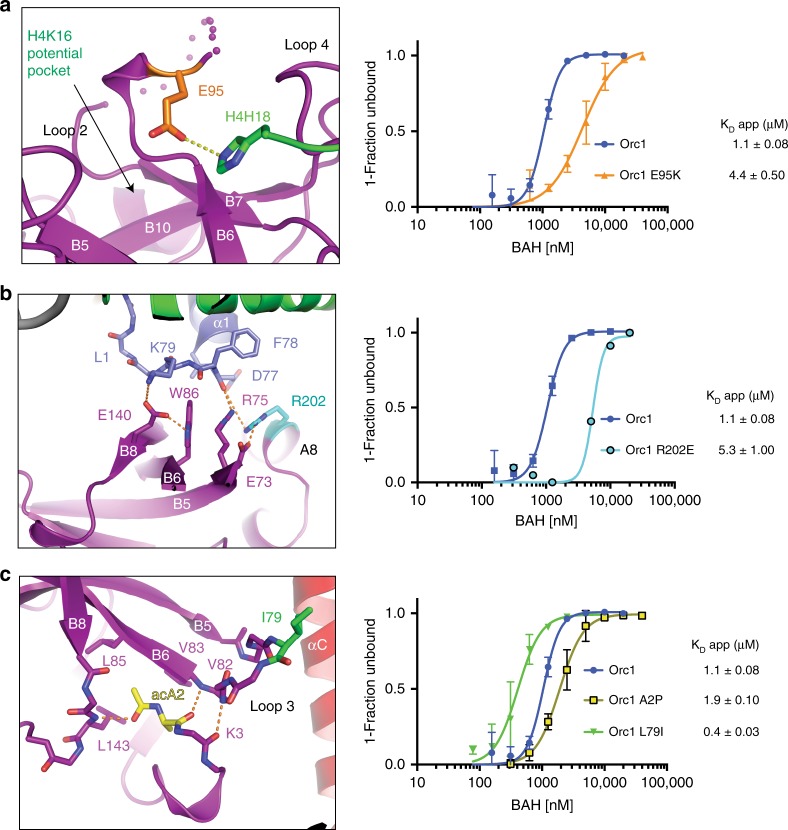
Functional characterization of Orc1-nucleosome interface. **a**–**c** Structural depiction of interactions between residues in Orc1 chosen for mutagenesis and nucleosomes (left panels), and the impact of these mutations on nucleosome binding measured by EMSA (right panels). **a** Orc1 E95 (orange) interacts with H18 of histone H4, and mutation of this residue to lysine (E95K) results in decreased affinity for the nucleosome as measured by EMSA. **b** Orc1 R202 makes a network of interactions that contribute to the stabilization of the LRS-interacting residues. The R202E mutation decreases the affinity of Orc1 for the nucleosome. **c** Acetylation of A2 at the N-terminus of Orc1 (yellow) helps to stabilize the conformation of Loop 3, and the L79I mutation (green) increases VdW interactions of the same Loop 3. A2P mutation, which prevents acetylation of the N-terminus of Orc1, results in decreased affinity for nucleosome. L79I has the opposite effect, resulting in an increased affinity of Orc1 for the nucleosome. Each data point and error bar represent the mean ± s.d. from three independent experiments. The standard errors of dissociation constants (*K*_D_) are indicated

To investigate the in vivo consequences of Orc1 BAH domain-nucleosome interactions, we analyzed meiotic DNA double-strand break (DSB) formation. Programmed DSB formation is essential for meiotic recombination but is suppressed in repetitive regions of the genome^61^. In yeast, the Orc1 BAH domain is necessary to suppress DSBs in the vicinity of ribosomal DNA (rDNA)^8^. To address whether direct nucleosome interactions of the Orc1 BAH domain with nucleosomes underlie its function at rDNA borders, we designed a panel of structure-guided modifications in Orc1 BAH that would either disrupt or strengthen nucleosome interactions. Specifically, we focused on E95K, R202E, A2P, and L79I mutations, to target different regions of the Orc1 BAH-nucleosome interface (Fig. 5a–c). E95 plays a central role in stabilizing histone H4 tail on the surface Orc1 BAH, where it interacts with H4H18 (Fig. 5a). Glutamic acid 95 replacement with lysine (E95K) generates a charge swap, reflected in a 4.0-fold loss of affinity (Fig. 5a and Supplementary Fig. 7) as shown by EMSA. Another tested residue, R202 is located at the beginning of helix A8, where its side chain is projected toward the LRS surface of the nucleosome (Fig. 5b). In the Orc1 structure presented here, the R202 side chain interacts with the main chain carbonyl of D77 of histone H3. Moreover, R202 could also play an interface-stabilizing role as its side chain makes a salt bridge with Orc1 E73, perhaps stabilizing the conformation of other Orc1 residues involved in LRS recognition including R75, W86, and E140 (Fig. 5b). Mutation of R202E would generate an electrostatic repulsion that could lead to loss of optimal LRS recognition by creating a disruption in the interaction network observed in the region (Fig. 5b). Indeed, this mutation results in a significant loss of affinity of Orc1 BAH for nucleosome when compared with the WT domain (4.9-fold decrease in affinity) (Fig. 5b and Supplementary Fig. 7). Mutation of alanine to proline (A2P) in the Orc1 BAH domain has been identified as a loss of function by a functional screen^20^. In our structure, as well as other structures of the BAH domain, A2 is acetylated at the α-amino group when purified from eukaryotic cells (Supplementary Fig. 8). The N-terminal residues A2 and K3 create anti-parallel hydrogen bonds with the backbones of residues V82 and V83 of β-strand B6 (Fig. 5c). These interactions lead to the stabilization of Loop 3 conformation that is optimal for interactions with nucleosomes (Fig. 5c). The A2P mutation does not support Orc1 acetylation by NatA, as confirmed by mass spectrometry analysis (Supplementary Fig. 8). As expected, nucleosome affinity for the A2P mutant showed roughly a twofold loss when compared with the WT domain (Fig. 5c and Supplementary Fig. 7). Finally, the L79I mutation in Orc1 BAH allowed us to generate a complex sufficiently stable biochemically for crystallization. This mutation (L79I) results in an increased affinity of Orc1 BAH for the nucleosome when compared with the WT domain (2.8-fold increase in affinity) (Fig. 5c and Supplementary Fig. 7). In summary, we validated our structure biochemically by showing the importance of selected residues in nucleosome binding and created a panel of Orc1 BAH mutants for in vivo studies.

**Fig. 6:**
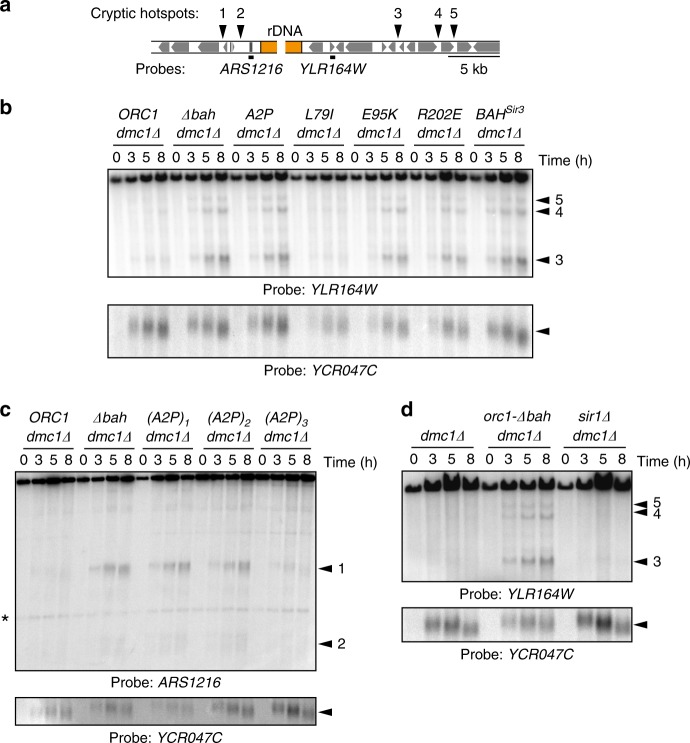
Orc1-nucleosome contacts protect rDNA borders during meiosis. **a** Schematic indicating probe locations next to the rDNA used in panels (**b**), (**c**), and (**d**), as well as location of cryptic hotspots with respect to gene bodies (gray boxes). **b** Effect of Orc1 BAH mutations. Southern blot of the right rDNA flank (probe *YLR164W*). The *dmc1Δ* mutation prevents DSB repair, allowing analysis of total DSB activity. The A2P, E95K, and R202E mutants experience strong DSB activity near the rDNA while L79I does not. Replacement of Orc1 BAH domain with that of Sir3 does not rescue the wt phenotype. DSB formation at the *YCR047C* locus serves as control for efficiency of meiotic induction. **c** Dosage effect of Orc1 A2P BAH mutation. Strains carry 1, 2, or 3 integrated copies of the *orc1-A2P* construct. Southern blot of the left rDNA flank (probe *ARS1216*). **d** Effect of *SIR1* deletion (probe *YLR164W*)

To test the functional relevance of direct Orc1 BAH-nucleosome interactions in vivo, we generated BAH mutant yeast strains with indicated mutations, and tested for the activation of cryptic DSB hotspots near the rDNA in synchronous meiotic cultures by Southern blotting (Fig. 6a). The E95K and R202E mutants experienced strong DSB activity near rDNA (*ARS1216*), similar to a BAH deletion (*orc1-Δbah*; Fig. 6b), indicating a loss of BAH function. A2P shows the same effect, and its effect is dose-dependent, where increasing the number of genomic copies of A2P BAH domain rescues the WT phenotype presumably by increasing the local concentration of Orc1 (Fig. 6c). These results are consistent with the reduced affinity of these mutants for the nucleosome, and suggest that DSB suppression by Orc1 requires the chromatin binding activity of the BAH domain. The L79I mutation, which increases the affinity of Orc1 BAH domain for the nucleosome, behaves like the WT protein (Fig. 6b), further supporting the notion that Orc1 BAH forms an important nucleosome interaction which when disrupted, results in DSB accumulation. Importantly, this Orc1 BAH domain activity is distinct from the BAH domain of Sir3 because substituting the BAH domain of Orc1 with Sir3 BAH causes a loss of function similar to a BAH deletion (Fig. 6b). In addition, the role of the Orc1 BAH domain in DSB suppression is distinct from its function in transcriptional silencing because DSB suppression does not require Sir1 (Fig. 6d). These data point to a novel chromatin role of the Orc1 BAH domain in meiotic prophase.

## Discussion

Chromatin conformation regulates all DNA-based processes in eukaryotic genomes. Specificity of binding within chromatin is regulated by many features of nucleosomes, including posttranslational histone modifications^62^. Our structural, biochemical, and functional data provide a first glimpse of an interface between the essential ORC complex and chromatin and show, at atomic resolution, how specific histone modifications regulate this interaction.

In recent years, BAH domains emerged as a class of nucleosome-binding domains with diverse histone modification specificities, often playing a role in epigenetic inheritance and gene regulation^44^. Orc1, an evolutionarily conserved subunit of the ORC complex, contains a BAH domain^9^. Here, we provide evidence that unlike its close homolog Sir3, Orc1 binds nucleosomes without having a strong preference for the acetylation state of H4K16. We hypothesize that it could bind equally well at euchromatin where K16 is acetylated, or at heterochromatin where K16 is unmodified. This unique feature would allow the ORC complex to bind chromatin across the genome, to perform its diverse functions. This lack of discrimination of K16 modification status by *S*. *cerevisiae* Orc1 is likely an evolutionary conserved feature given the conservation of ORC function in all eukaryotes^63^. Also, we hypothesize that during evolution, with the emergence of novel histone modifications to demarcate the genome, the Orc1 BAH developed the ability to bind novel histone modifications (such as H4K20me2)^36^ and both unmodified and acetylated H4K16. Both the mouse and human Orc1 BAH domains bind with high specificity to H4K20me2 peptides without a strong preference for the K16 acetylation status^36^. These data would have to be further quantitatively and structurally corroborated on nucleosome substrates. Due to low overall conservation with yeast Orc1 BAH domain (11% sequence identity between mouse and yeast Orc1 BAH domains), it is currently difficult to model the interaction of the mammalian Orc1 BAH domain bound to the nucleosome.

While the ORC complex binds many regions in the yeast genome, the full spectrum of loci where its direct interactions with nucleosomes play important roles is not known. At silencers, Orc1 binds DNA, but it is not clear if it binds directly to nucleosomes. Orc1 has also been shown to physically interact throughout internal regions of *HMR* and silencers, which could point to a function distinct from the initiation of silencing and could involve direct nucleosome interactions^64^. Similarly, at telomeres, Orc1 could bind to nucleosomes to maintain repressive chromatin structure. In line with this hypothesis, we show here that A2P mutation, which prevents Orc1 acetylation by NatA-Ard and was previously shown to impair *HM* and telomeric silencing^20^, lowers the affinity of Orc1 for nucleosomes. Mutations reported to impact replication at a specific set of origins result in compromised Orc1-nucleosome binding^11^, suggesting that ORC nucleosome interactions are required for the function of these origins. In addition, our data point to a novel chromatin role of the Orc1 BAH domain in meiotic prophase. We show that mutations that directly disrupt, but not those that stabilize Orc1-nucleosome interactions in vitro, result in accumulation of DSBs at the borders of rDNA locus during meiosis in vivo. It is tempting to speculate that boundaries between heterochromatin and euchromatin such as those found at the edges of the rDNA use a specific border-control mechanism where (at least in yeast) the direct interactions between Orc1 and nucleosomes, as mediated by BAH domain, are essential. We demonstrate that this protective function of ORC is decoupled from the activity of Sir1 which is strictly required for silencing at the *HM* loci^14^. DSB induction near the rDNA is dependent on the catalytic activity of the histone deacetylase Sir2^8^, which primarily targets H4K16ac. It is possible that binding of the Orc1 BAH domain protects acetylated nucleosomes from Sir2 activity, thereby directly or indirectly suppressing meiotic DSB activity. If true, this would explain why the Orc1 BAH domain but not the Sir3 BAH domain can support the integrity of these loci.

## Methods

### Strains and plasmids

A fragment corresponding to *S*. *cerevisiae* Orc1 amino acids 1–214 as well as Sir3 BAH (1–214) were subcloned with a C-terminal His-tag into pACEBAC1 vector by Gibson Assembly (NEB) for Baculovirus expression. ORC1 was cloned into pRS406 for yeast expression. All point mutants were introduced by Q5 site-directed mutagenesis (NEB) and verified by sequencing. All yeast strains are of the SK1 background, and are listed in Supplementary Table 2. For genomic integration, ORC1 constructs were linearized with StuI to target them to the *ura3* locus. Copy number of the integrated constructs was determined by Southern blotting of total genomic DNA digested with BglII using the integration construct as probe. All strains carry a single copy of the respective construct unless indicated otherwise. Bacterial expression vectors of *X. laevis* histones H2A, H2B, H3, and H4 were a gift from Dr. Karolin Luger, and 147 bp 601 DNA plasmid was generated by Genscript. Plasmid pASB567_9g7 (pCDF-PylT-AraP-His6-H3(∆93–98)-TEV-H4K16 amber codon expression vector) was provided by Dr. Heinz Neuman (MPI Dortmund). The pAcKRS-3 plasmid containing acetyl-Lysyl-tRNA sythetase/tRNA_cua_ was provided by Dr. J. Chin (MRC, UK). The E. coli strain C321.ΔA.exp was obtained from Addgene (#49018).

### Protein expression

Wild-type Orc1 (1–214) BAH domain, Sir3 BAH (1–214) and mutants were expressed in SF9 insect cells for 36 h at 27 °C. After expression, cell pellets were lysed with cell homogenizer in Ni-binding buffer containing 25 mM Tris pH 8.0, 500 mM NaCl, 5% glycerol, 5 mM imidazole, 2 mM β-mercapthoethanol, and EDTA-free protease inhibitor mix (Roche). Clarified lysate was applied to Nickel affinity chromatography (Ni-NTA, Qiagen) and equilibrated with Ni-binding buffer. After washing with Ni-binding buffer containing 50 mM imidazole, proteins were eluted with the same buffer containing 300 mM imidazole. The eluted fractions were desalted, and further purified on SP-Sepharose FF (GE Healthcare), and Superdex-200 (GE Healthcare). Peak fractions were concentrated and stored at −80 °C in storage buffer (10 mM HEPES pH 7.5, 150 mM NaCl, 5 mM DTT). The genetically encoded histone H4K16ac was produced according to Wilkins et al.^51^. Briefly, E. coli C321.ΔA.exp cells were transformed with pAcKRS-3 and pASB567_9g7. Transformed bacteria were grown in LB broth supplemented with 50 μg/ml kanamycin and 75 μg/ml spectinomycin and 20 mM nicotinamide at 37 °C, until the culture reached OD600 ~0.8. In total, 10 mM *N*_*ε*_-Acetyl-L-lysine (Sigma) was subsequently added, and protein expression was induced by addition of 0.2% arabinose (Sigma). Cells were harvested 16 h after induction. The histone His6-H3(∆93-98)-H4 K16ac fusion was purified from inclusion bodies, through Nickel affinity purification after solubilization with 6 M guanidinium hydrochloride (Sigma). The His6-H3(∆93-98) tag was cleaved with TEV protease. Finally, the histone prep was dialyzed against 5 mM β-mercaptoethanol, and lyophilized for further use. The histone H4 modifications were verified by western blot using anti-Histone H4 acetyl Lys16 (Active Motif, #39167) and anti-Histone H4 Abcam (ab7311) was used for the loading control (Supplementary Fig. 6A).

### Nucleosome reconstitution

Nucleosomes were reconstituted as described^65^. Briefly, lyophilized histones were dissolved in unfolding buffer, mixed in equimolar ratio and dialyzed against refolding buffer to form the histone octamer. Properly folded octamer was purified by gel filtration chromatography on a Superdex 200 26/600 (GE Healthcare). 147 bp Widom-601 DNA fragment was released from a plasmid by EcoRV followed by PEG precipitation. Nucleosomes were  assembled using a gradient dialysis technique by mixing equimolar amounts of histone octamer with DNA in refolding buffer. Nucleosomes were further purified by ion exchange chromatography on Resource Q column (GE Healthcare). The nucleosome concentration was estimated by absorbance at 260 nm. The quality of the nucleosome prep was evaluated by native PAGE.

### Orc1/nucleosome complex assembly

The complex was reconstituted by mixing Orc1 BAH L79I mutant with nucleosome in 3:1 molar ratio and dialyzed overnight against 10 mM Tris pH 7.5, 25 mM sodium acetate (NaAc) and 2 mM DTT. The complex was further purified by gel filtration using Superdex 200 10/300 column equilibrated with 10 mM Tris pH 7.5, 50 mM NaAc, and 2 mM DTT. The purified complex was concentrated with a 10 K MWCO centrifugation device (Amicon), and its concentration was estimated by absorbance at 260 nm.

### Protein crystallization

Orc1 BAH L79I/nucleosome complex was concentrated to 3 mg/ml in a solution containing 10 mM Tris pH 7.5, 12 mM NaAc, and 2 mM DTT. The crystals were grown at 4 °C by using the sitting drop vapor diffusion method, where equal volumes of complex and crystallization solution containing 6 mM Na-Cacodylate pH 6.0, 0.4 mM Spermine-HCl, 2 mM MgCl_2_, and 1.75% v/v PEG 400 were mixed. All crystals were cryoprotected in crystallization solution by a stepwise increase of PEG400 (40–50%) and frozen in liquid N_2_.

### Structure determination and refinement

Diffraction data were collected at 110 K from single crystals at Advanced Photon Source (APS; NE-CAT beamline 24-ID) at an X-ray wavelength of 0.9791 Å with a Dectris Pilatus 6M-F detector. Data were processed using HKL-2000 and CCP4 packages. The structure was solved by molecular replacement (MR) using the nucleosome core particle (PDB ID: 3LZ1)^66^ and the apo Orc1 BAH (PDB ID: 1M4Z)^19^ as search models by the program PHASER^67^. After MR, the DNA model was replaced with the 147 bp Widom-601 from the Sir3 BAH domain in complex with a nucleosome structure (PDB ID: 3TU4)^31^, to match our DNA sequence. Crystallographic refinement was carried out using Phenix^68^ and model building with COOT^69^. All structure figures were generated with PyMOL (Schrödinger).

### Nucleosome-binding assay

Increasing amounts of Orc1 or Sir3 proteins were incubated with 50 or 25 nM nucleosomes in reaction buffer (10 mM Tris pH 7.5, 50 mM NaCl, 2.5% glycerol, and 1 mM DTT) at room temperature for 30 min. The binding reactions were resolved on native polyacrylamide gels (6% (for Sir3) or 8% (for Orc1) PAGE, 0.2 X TBE), stained with ethidium bromide (Bio-Rad), visualized on a Typhoon Trio + scanner (Molecular Dynamics), and quantified using the program ImageQuant 5.2 v (Molecular Dynamics). 100 bp Plus DNA ladder (GoldBio) was used to monitor the run. The amount of Orc1 or Sir3 bound to nucleosomes was determined by measuring the decrease in free nucleosome in each reaction. The background was subtracted from BAH-free samples. The free DNA was taking under consideration for the calculation of free nucleosomes. The apparent *K*_D_ and the Hill coefficient for each binding curve was calculated by fitting the specific binding with the Hill slope equation using the program Prism 7 (GraphPad). The final parameters were calculated from at least three independent experiments (*n* ≥ 3/data point). Data were plotted as mean ± s.d. A representative EMSAs from each experiment are presented in Supplementary Figs 6 and 7.

### ChIP-seq data analysis

The Orc1 (2014) ChIP-seq data from sonicated samples were downloaded from SRP034921^52^. The MNase H3 and H4K16ac ChIPseq dataset used was generated by Nir Friedman’s group^53^. The ChIP-seq data were aligned as follows. First, we identified the ARS consensus sequences in the annotated ARS regions downloaded from UCSC Genome Browser. These origins were aligned by centering the sequences on the ARS consensus sequences using the same strategies as previously described^55^. We also oriented the T-rich strands of each ARS as the top strand. Next, we selected ARS sites that showed ORC binding based on the ORC ChIP-seq dataset (SRP041314) generated by MacAlpine’s group^54^. These origins were aligned by centering the sequences on the ARS consensus sequences. To evaluate ARS alignment strategies, we used the previously annotated ARS origins. Orc1 peaks were detected by MACS. ARS-overlapping Orc1 peaks were identified by the function findOverlaps in R and each peak was divided into 20 consecutive 50 bp windows centered at the first nucleotide of the ACS with T-rich strands on the top (forward) strand, similar to the procedure in DynaMO. Orc1, H4K16ac, and H3 signals at these 50 bp windows were calculated using the function countOverlaps in R. The Orc1, H4K16ac, and H3 signals were plotted as a heatmap. Orc1 peaks were ordered from top to bottom by the distances between the summits of Orc1 peaks and the closest ends of the chromosomes.

### Synchronous meiosis

Strains were induced to undergo synchronous meiosis as described^70^. Briefly, strains were pre-grown for 24 h in YPD, diluted at OD_600_ = 0.3 in BYTA pre-sporulation medium, and grown for another 16.5 h. To induce meiosis, cells were washed twice with water and resuspended OD_600_ = 1.9 in 0.3% potassium acetate at 30 °C. At the indicated time points, cells were killed with 0.1% sodium azide, washed once with TE, and frozen.

### Southern analysis

Genomic DNA for DSB hotspot analysis was purified from spheroplasts as described^71^. Samples were digested with HindIII (for probing with *ARS1216* or *YCR047C*) or ApaLI (for probing *YLR164W*) and separated in a 0.6% agarose/1X TBE gel for 18–20 h. Samples were blotted onto Hybond-XL membranes (GE Healthcare) using alkaline transfer and probed using previously described probes^8^. Hybridization signal was detected using a Typhoon FLA9000.

### Mass spectrometry analysis of Orc1

The purified proteins were infused into the Orbitrap Fusion Lumos mass spectrometer at a concentration of 1 pmol/µl in 40% acetonitrile and 0.5% acetic acid using a 360 × 75 µm fused silica line. The MS1 spectra were acquired in the Orbitrap with a resolution of 240,000, source fragmentation energy of 40% and a scan range of 400–2000. For each scan five microscans were averaged. All MS2 scans were acquired in the Orbitrap with a resolution of 240,000, an isolation window of 5 Da, and an ETD reaction time of 10 ms. The intact mass was deconvoluted using Intact Mass™ (Protein Metrics) and the MS/MS spectra were searched using Prosight Light software against the provided sequence and manually verified.

### Reporting summary

Further information on research design is available in the Nature Research Reporting Summary linked to this article.

## Supplementary information

Supplementary Information

Reporting Summary

Source Data

## Data Availability

All materials will be available from the corresponding authors upon a reasonable request. Atomic coordinates and structure factors have been deposited in the Protein Data Bank under accession number 6OM3. The source data underlying Figs 3 a, b, d, e, 4b, d, e, 5a–c, 6 b–d, and Supplementary Figs 6 and 8 are provided as a Source Data file.
