# Supplementary material for: Structure and function of the Orc1 BAH-nucleosome complex
Source: Nat Commun. 2019 Jul 1;10:2894. doi: 10.1038/s41467-019-10609-y (PMC6602975; doi:10.1038/s41467-019-10609-y)
Supplement: Supplementary file 1 — Supplementary Information [file 41467_2019_10609_MOESM1_ESM.pdf]

Supplementary Information for

Structure and function of the Orc1 BAH-nucleosome complex

Pablo De Ioannes, Victor A. Leon, Zheng Kuang, Miao Wang, Jef D. Boeke, Andreas Hochwagen and Karim-Jean Armache*

*The correspondence should be addressed to K-JA (karim-jean.armache@med.nyu.edu)

This PDF file includes

Supplementary Notes

Figs. S1 to 8

Supplementary Table 1 and 2

Supplementary Notes

Additional information to structure determination

The complex was reconstituted by mixing 3:1 excess of Orc1 BAH over nucleosome, and purified by gel filtration. The crystals were grown at 4°C, and diffracted synchrotron radiation anisotropically to 4.5 Å. Post-crystallization stabilization treatment reduced anisotropy and allowed collection of complete diffraction data to 3.3 Å (Table 1 and supplementary methods). A molecular replacement solution revealed two nucleosome copies in the asymmetric unit (Supplementary Fig. 1A). A positive difference electron density for BAH domain was found on both sides of one of the nucleosomes, while the other complex only showed one well-ordered copy of BAH (Supplementary Fig. 1B). A model of the free Orc1 BAH domain structure (PDB ID: 1M4Z) was fitted into these densities, and several rounds of rebuilding and refinement were performed. The final model presents an excellent stereochemistry and R/R_{free} of 21.30 / 24.97 (Supplementary Table 1).

Additional information to general architecture of the complex

The structure presents a novel packing with two complexes in the asymmetric unit (Supplementary Fig. 1). The three ordered copies of the BAH domains are very similar (RMSD=0.60 Å) and show the typical architecture that consists of a central anti-parallel beta sheet (B4-B11) which connects to the independent beta subdomain (B1-B4), and the C-terminal helices (A7 and A8). The H domain, which has been implicated in Sir1 binding and consequently is essential for SIR complex recruitment to the mating-type locus, is located between strands B6 and B7 (Fig. 1 and Supplementary Fig. 2). The four large loops that connect secondary structure elements (L1 between B1 and B2, L2 between B4 and B5, L3 between B5 and B6 and L4 between B10 and B11) are well ordered in the structure and project into the nucleosome surface. In general, we observe good model coverage for the BAH domains, except for the H domain which is partially disordered.

Supplementary figures.

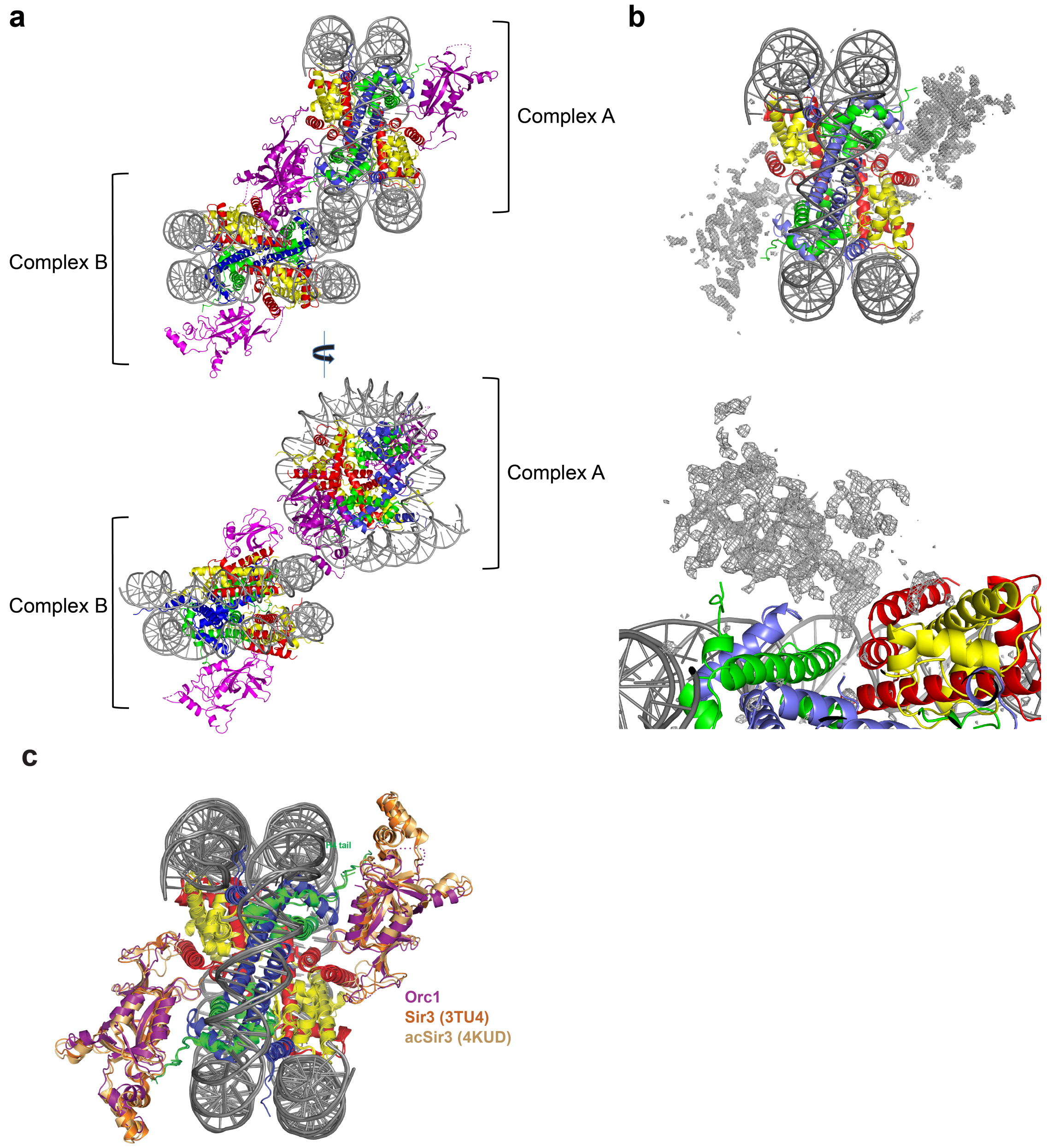

Supplementary Figure 1. Overview of the asymmetric unit and MR solution. a Asymmetric unit of Orc1 BAH L79I-Nucleosome complex. Front view (Top). Side view (Bottom). **b** Fo-Fc electron density map from a MR solution (using only nucleosome as a search model) contoured at 2.5 σ. Front view (Top). Detailed side view of Orc1 BAH density (Bottom). **c** Structural alignment between Orc1 (purple) versus unacetylated Sir3

(orange) (PDB ID: 3TU4) and acetylated Sir3 (wheat) (PDB ID: 4KUD) nucleosome complexes.

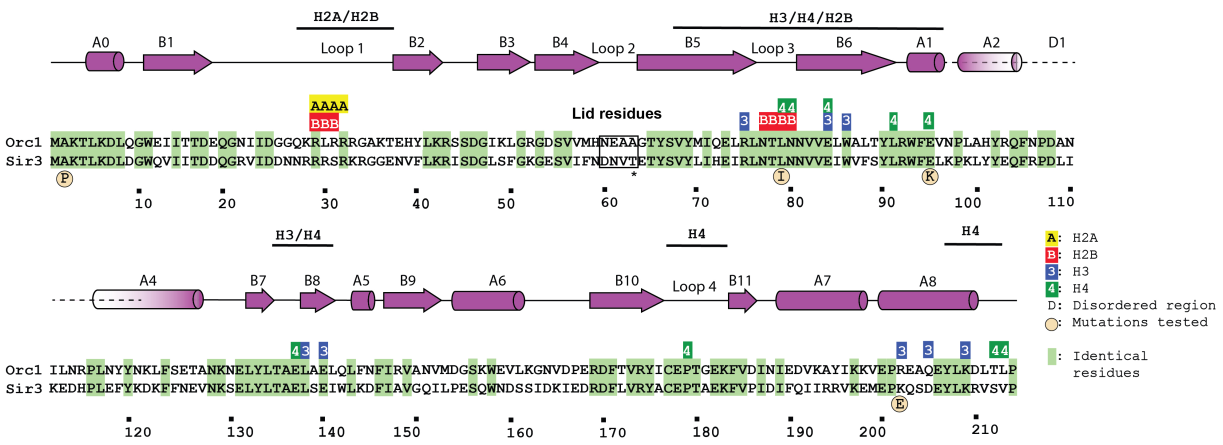

Supplementary Figure 2. Sequence alignment of Orc1 BAH and Sir3 BAH. Colored squares below the secondary structure indicate the corresponding interacting histones with residues in Orc1 BAH domain (within 4.1 Å). Residues mutated for biochemical experiments are highlighted and displayed as spheres below the alignment. Loop 2 Lid residues are highlighted in a box. Asterisk represents a dominant loss of silencing mutation in Sir3 Lid.

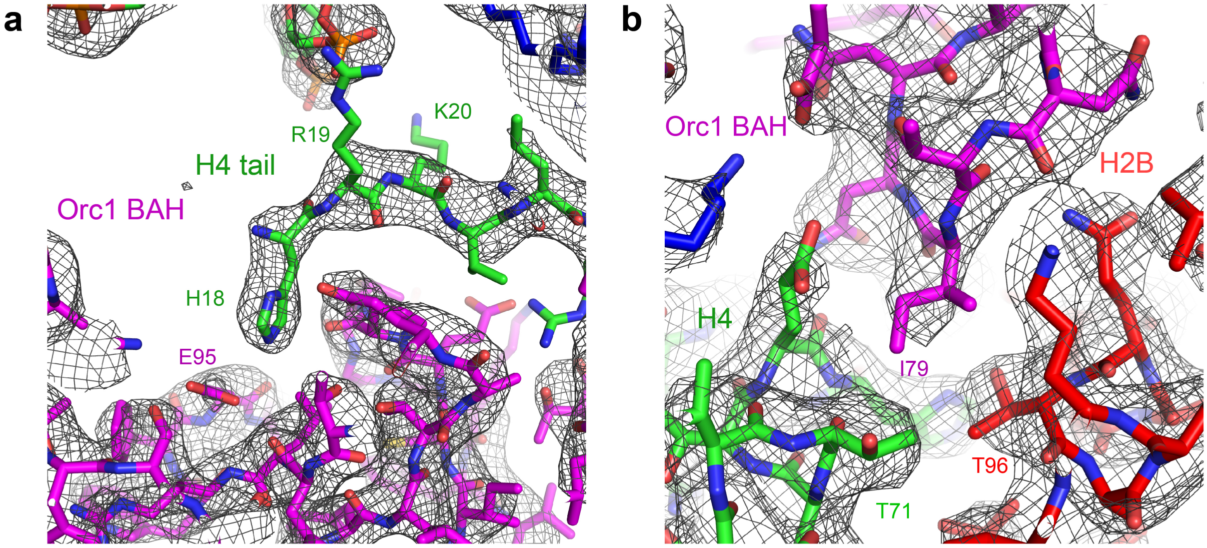

Supplementary Figure 3. Electron density maps. a Maximum-likelihood 2Fo-Fc electron density map contoured at 1.0 σ for Orc1 BAH / Histone H4 interface. **b** Orc1 BAH Loop 3 / nucleosome interface.

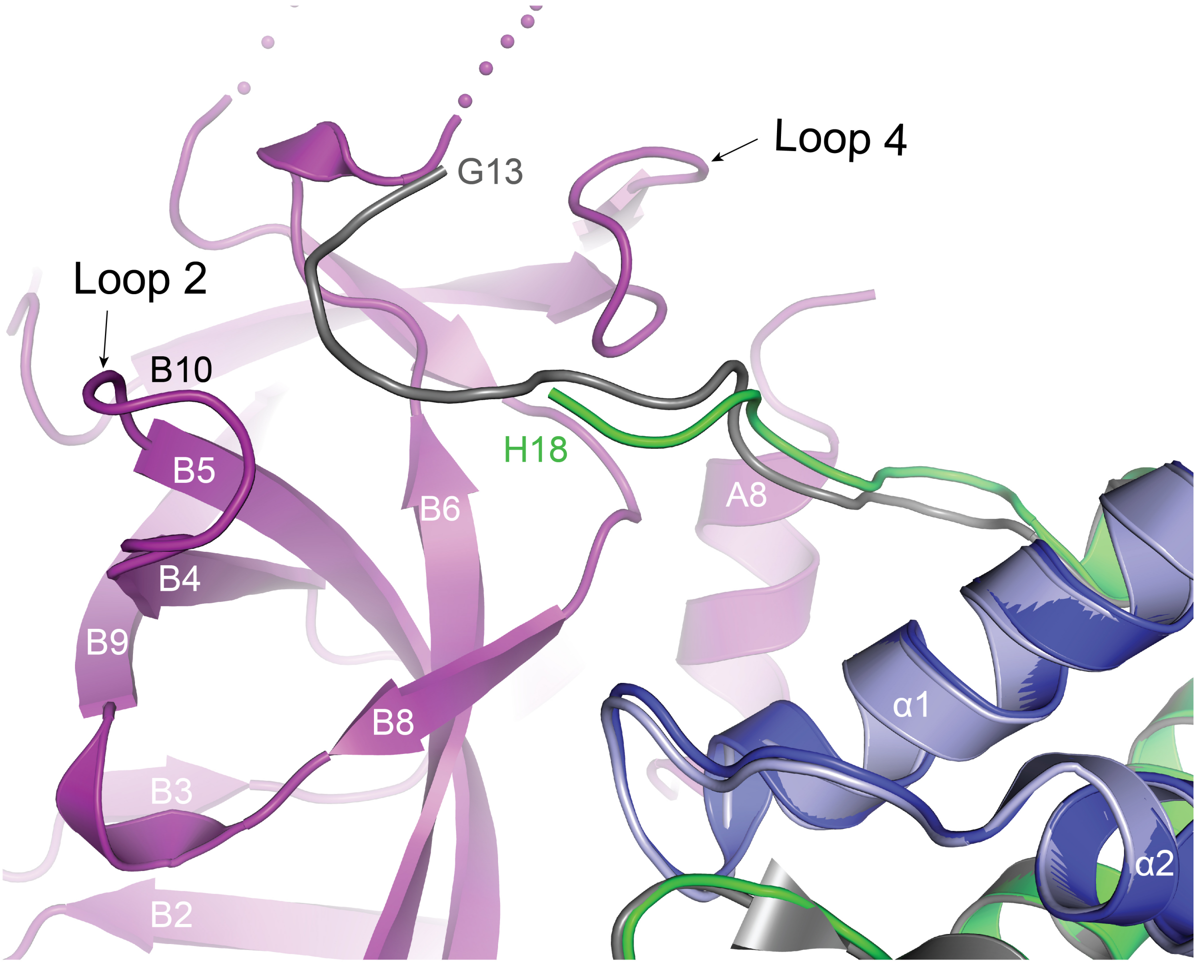

Supplementary Figure 4. **Structural comparison of H4 tail recognition in Orc1 and Sir3 BAH/nucleosome complexes.** Detailed view of H4 tail recognition region in Orc1 (purple). H4 tail from Orc1 structure is depicted in green and H4 tail from acetylated Sir3 structure in gray (PDB ID: 4KUD).

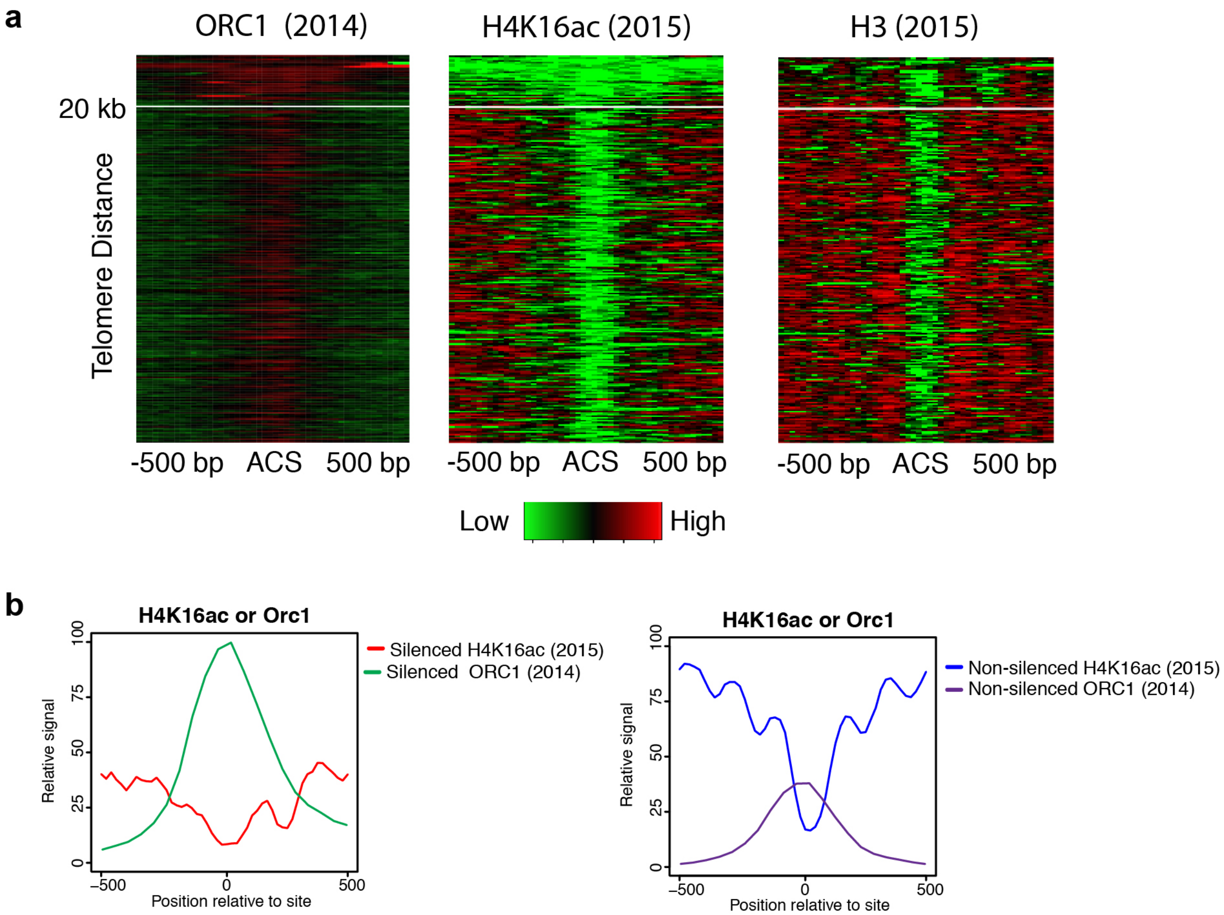

Supplementary Figure 5. Orc1 localization and acetylation state of H4K16 at ACS. **a** Heatmap showing the spatial distribution of Orc1 binding signals, and H4K16ac or H3 at ARS-overlapping Orc1 peaks. Orc1 peaks are centered by aligning the first nucleotide of the ACS in the center and orienting T-rich strands on the top (forward) strand[1, 2] and ranked by the distances between Orc1 peak summits and the closer chromosome ends. Orc1 peaks with distances less than 20 kb (top panel) and peaks with distances of more than 20 kb (bottom panel) are shown. **b** Averaged Orc1 (2014) and H4K16ac (2015) at silenced or non-silenced origins.

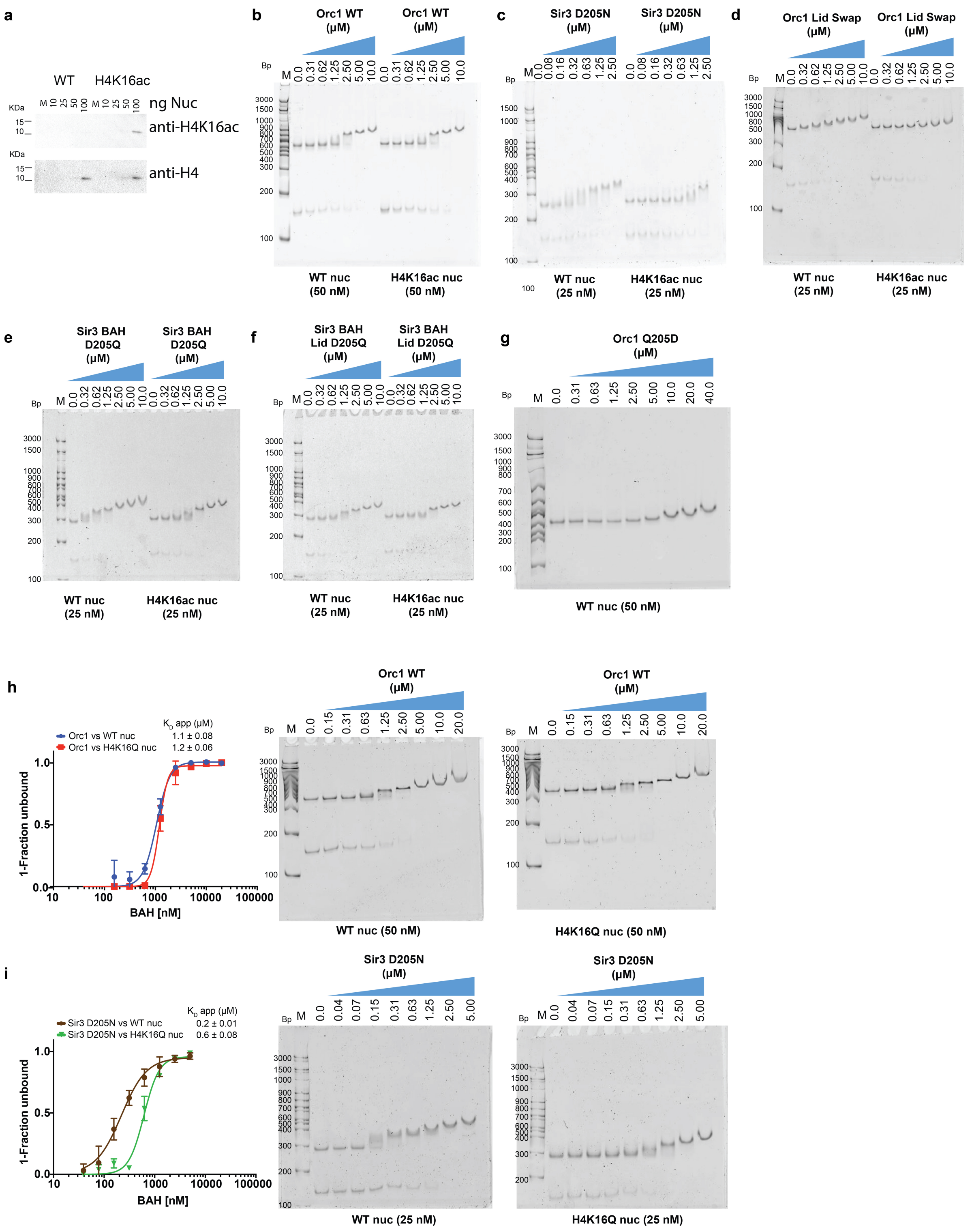

Supplementary Figure 6. Western blot of the H4K16ac nucleosome and representative EMSAs of BAH domains binding to nucleosomes. a Western blot analysis of H4K16ac nucleosome. Representative EMSAs of **b** Orc1 WT vs unmodified

(WT nuc) or H4K16ac nucleosome (H4K16ac nuc), **c** Sir3 D205N vs WT nuc or H4K16ac nuc, **d** Orc1 Lid Swap vs WT nuc or H4K16ac nuc, **e** Sir3 D205Q vs WT nuc or H4K16ac nuc, **f** Sir3 Lid Swap D205Q vs WT nuc or H4K16ac nuc, **g** Orc1 Q205D vs WT nuc, **h** Quantification of EMSAs and representative gels of Orc1 WT vs WT nuc or H4K16Q (acetyl mimic) nucleosomes, **i** Quantification of EMSAs and representative gels of Sir3 D205N vs WT nuc or H4K16Q nuc. Each data point and error bar represents the mean ± s.d. from three or more independent experiments. The standard errors of each dissociation constants (K_D) are indicated.

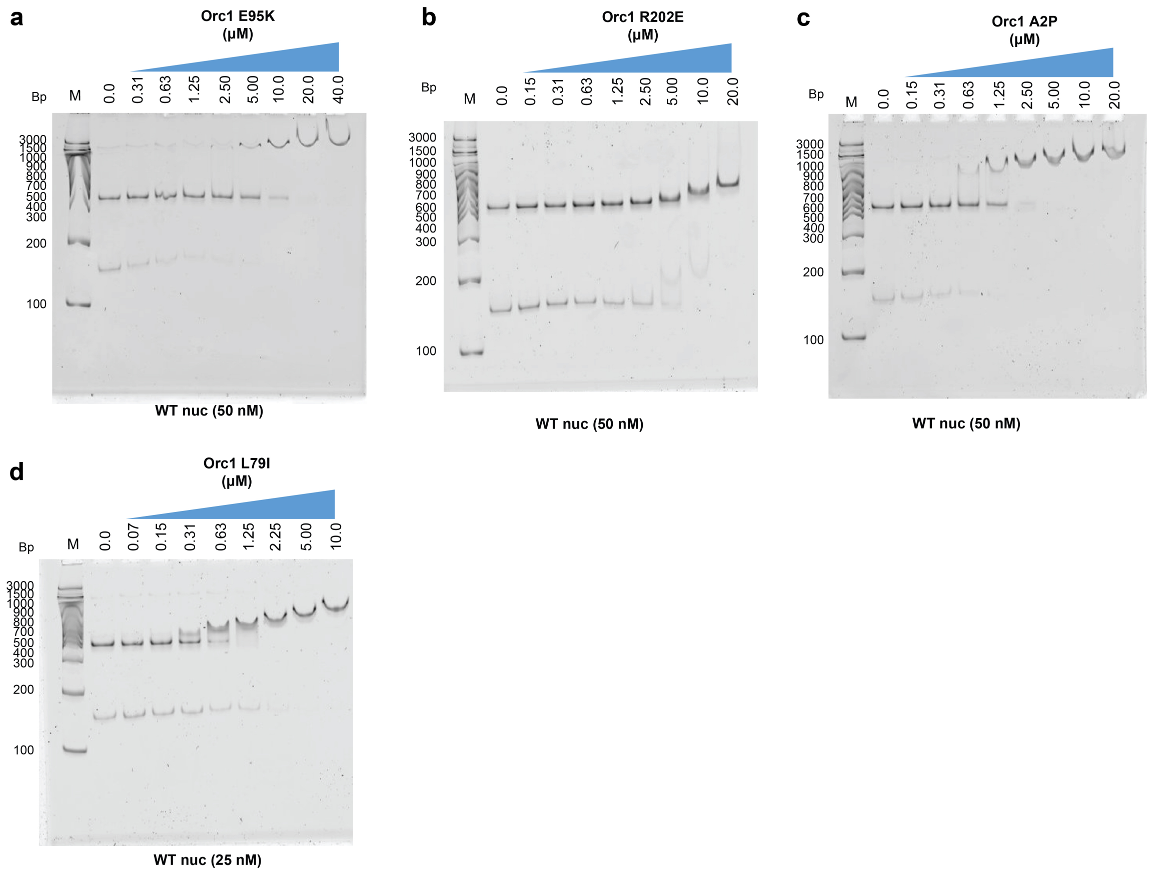

Supplementary Figure 7. Representative EMSAs of Orc1 BAH domain mutants binding to nucleosomes. a Orc1 BAH E95K vs unmodified nucleosome (WT nuc). **b** Orc1 BAH R202E vs WT nuc. **c** Orc1 BAH A2P vs WT nuc. **d** Orc1 BAH L79I vs WT nuc.

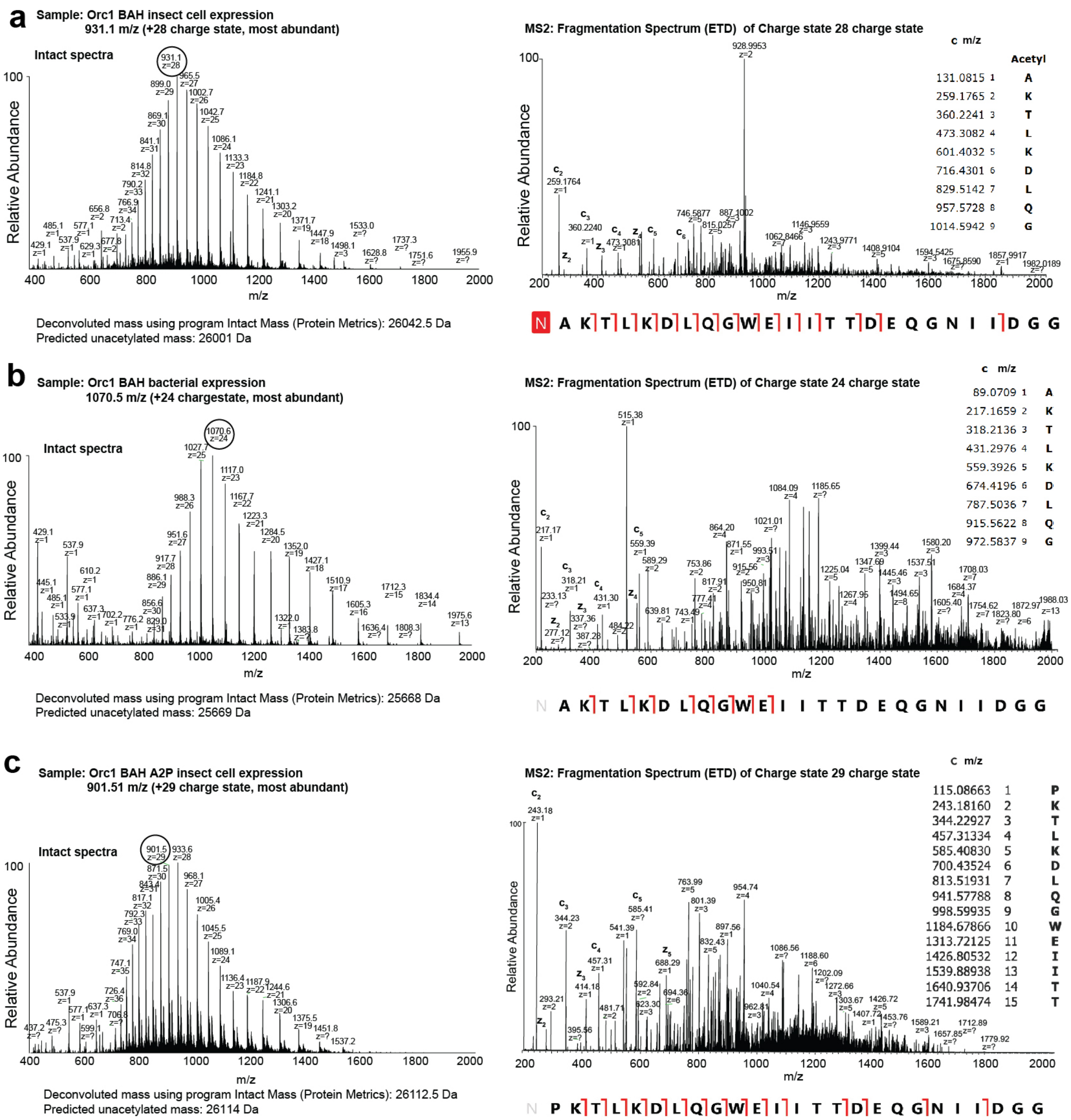

Supplementary Figure 8. Mass spectrometry analysis of Orc1 samples. The MS1 spectra of intact protein (left). The MS2 spectra were searched using Prosight Light and manually verified. Singly charged fragment ions containing the N-terminus (c ions) and the C-terminus (z ions) are indicated in the spectra (right). Predicted m/z for c fragments are shown in the table. N-terminus fragment ion coverage is shown under the MS2 spectra. **a.** Orc1 BAH WT expressed in insect cells. Shown is the ETD MS/MS spectrum of 931.1 m/z (+28 charge state) of intact Orc1 BAH WT. The insert shows the MS1

spectrum (averaged 100 scans). The protein MW is 26042.5 Da consistent with an acetylated Orc1 BAH WT and the N-terminal fragment ions localize the acetylation to the N-terminus. **b.** Orc1 BAH expressed in insect cells. Shown is the ETD MS/MS spectrum of 1070.5 m/z (+24 charge state) of intact Orc1 BAH expressed in bacterial cells. The insert shows the MS1 spectrum (averaged 100 scans). The protein MW is 25668 Da consistent with an unmodified Orc1 BAH and the N-terminal fragment ions verify that the N-terminus is unmodified. **c.** Orc1 BAH A2P expressed in insect cells. Shown is the ETD MS/MS spectrum of 901.5 m/z (+29 charge state) of intact Orc1 BAH A2P expressed in bacterial cells. The insert shows the MS1 spectrum (averaged 100 scans). The protein MW is 26112.5 Da consistent with an unmodified Orc1 BAH A2P and the N-terminal fragment ions verify that the N-terminus is unmodified. Protein sequences of MS samples and sequences coverages are included in the source data.

Supplementary Table 1. Data collection and refinement statistics

	Orc1 BAH L79I–NCP WT
**Data collection**	APS 24ID-C (HKL-200)
Space group	$P2_1$
Cell dimensions	
a, b, c (Å)	100.25, 166.43, 168.28
α, β, γ (°)	90.00, 90.28, 90.00
Resolution (Å)	49.1 - 3.3 (3.36-3.30)
Rsym or Rmerge	0.117 (0.837)
I / σI	8.99 (1.40)
Completeness (%)	97.81 (97.88)
CC1/2	0.993 (0.506)
Redundancy	3.7
**Refinement**	
Resolution (Å)	49.1 - 3.30
No. reflections	81181
Rwork / Rfree(%)	21.30 / 24.97
No. atoms	28906
Protein	16894
DNA	12012
Ligand/ion	0
Water	0
B-factors	133.40
Protein	114.77
DNA	159.27
Ligand/ion	N/A
Water	N/A
R.m.s. deviations	
Bond lengths (Å)	0.003
Bond angles (°)	0.555

One crystal was used for each structure determination. *Values in parentheses are for highest-resolution shell.

Supplementary Table 2. Genotypes of yeast strains used in this study

Strain No.	Relevant genotype	Reference	Figure
NKY1455	*MATalpha, ho::LYS2, lys2, leu2::hisG, his4B::LEU2, MATa, ho::LYS2, lys2, leu2::hisG, his4X::LEU2-URA3, ura3, arg4-Bgl2, dmc1Δ::ARG4 ura3, arg4-Nsp, dmc1Δ::ARG4*	[3]	6d
H5865	*MATalpha, ho::LYS2, lys2, leu2::hisG, his4B::LEU2,*<br>*MATa, ho::LYS2, lys2, leu2::hisG, his4B::LEU2,*<br>*arg4-Bgl2(?), dmc1Δ::ARG4,*<br>*arg4-Bgl2, dmc1Δ::ARG4,*<br>*orc1::TRP1, ura3::orc1ΔNTD(1-235)::URA3*<br>*orc1::TRP1, ura3::orc1ΔNTD(1-235)::URA3*	[4]	6d
H6028	*MATalpha, ho::LYS2, lys2, leu2::hisG, his4B::LEU2,*<br>*MATa, ho::LYS2, lys2, leu2::hisG, his4B::LEU2,*<br>*ura3, dmc1Δ::ARG4, sir1Δ::hphMX4*<br>*ura3, dmc1Δ::ARG4, sir1Δ::hphMX4*	This study	6d
H9394	*MATalpha, ho::LYS2, lys2, leu2::hisG, his3::hisG,*<br>*MATa, ho::LYS2, lys2, leu2::hisG, his3::hisG, trp1::hisG, dmc1Δ::HIS3, orc1::TRP1, trp1::hisG, dmc1Δ::HIS3, orc1::TRP1, ura3::ORC1::URA3/ ura3::ORC1::URA3*	This study	6b, c
H9397	H9394 but *ura3::orc1-A2P::URA3(3x)/ ura3::orc1-A2P::URA3(3x)*	This study	6c
H9400	H9394 but *ura3::orc1-E95K::URA3/ ura3::orc1-E95K::URA3*	This study	6b
H9409	H9394 but *ura3::orc1-R202E::URA3/ ura3::orc1-R202E::URA3*	This study	6b
H9412	H9394 but *ura3::orc1-Δbah::URA3/ ura3::orc1-Δbah::URA3*	This study	6b, c
H9415	H9394 but *ura3::orc1-BAH^{Sir3}::URA3/ ura3::orc1-BAH^{Sir3}::URA3*	This study	6b
H9601	H9394 but *ura3::orc1-A2P::URA3/ ura3::orc1-A2P::URA3*	This study	6b, c
H9602	H9394 but *ura3::orc1-L79I::URA3/ ura3::orc1-*	This study	6b

	L79I::URA3		
H9713	H9394 but *ura3::orc1-A2P::URA3(2x)/ ura3::orc1-A2P::URA3(2x)*	This study	6c

Supplementary References

1. Belsky JA, MacAlpine HK, Lubelsky Y, Hartemink AJ, MacAlpine DM. Genome-wide chromatin footprinting reveals changes in replication origin architecture induced by pre-RC assembly. *Genes Dev* **29**, 212-224 (2015).

2. Eaton ML, Galani K, Kang S, Bell SP, MacAlpine DM. Conserved nucleosome positioning defines replication origins. *Genes Dev* **24**, 748-753 (2010).

3. Bishop DK, Park D, Xu L, Kleckner N. DMC1: a meiosis-specific yeast homolog of E. coli recA required for recombination, synaptonemal complex formation, and cell cycle progression. *Cell* **69**, 439-456 (1992).

4. Vader G, Blitzblau HG, Tame MA, Falk JE, Curtin L, Hochwagen A. Protection of repetitive DNA borders from self-induced meiotic instability. *Nature* **477**, 115-119 (2011).
