# Supplementary material for: Structure and function of the Orc1 BAH-nucleosome complex
Source: Nat Commun. 2019 Jul 1;10:2894. doi: 10.1038/s41467-019-10609-y (PMC6602975; doi:10.1038/s41467-019-10609-y)
Supplement: Supplementary file 2 — Reporting Summary [file 41467_2019_10609_MOESM2_ESM.pdf]

nature research

Corresponding author(s): Karim-Jean Armache

Last updated by author(s): May 6, 2019

Reporting Summary

Nature Research wishes to improve the reproducibility of the work that we publish. This form provides structure for consistency and transparency in reporting. For further information on Nature Research policies, see Authors & Referees and the Editorial Policy Checklist.

Statistics

For all statistical analyses, confirm that the following items are present in the figure legend, table legend, main text, or Methods section.

n/a	Confirmed	
☐	☒	The exact sample size (*n*) for each experimental group/condition, given as a discrete number and unit of measurement
☐	☒	A statement on whether measurements were taken from distinct samples or whether the same sample was measured repeatedly
☒	☐	The statistical test(s) used AND whether they are one- or two-sided<br>*Only common tests should be described solely by name; describe more complex techniques in the Methods section.*
☒	☐	A description of all covariates tested
☒	☐	A description of any assumptions or corrections, such as tests of normality and adjustment for multiple comparisons
☐	☒	A full description of the statistical parameters including central tendency (e.g. means) or other basic estimates (e.g. regression coefficient) AND variation (e.g. standard deviation) or associated estimates of uncertainty (e.g. confidence intervals)
☒	☐	For null hypothesis testing, the test statistic (e.g. *F*, *t*, *r*) with confidence intervals, effect sizes, degrees of freedom and *P* value noted<br>*Give P values as exact values whenever suitable.*
☒	☐	For Bayesian analysis, information on the choice of priors and Markov chain Monte Carlo settings
☒	☐	For hierarchical and complex designs, identification of the appropriate level for tests and full reporting of outcomes
☒	☐	Estimates of effect sizes (e.g. Cohen's *d*, Pearson's *r*), indicating how they were calculated

Our web collection on statistics for biologists contains articles on many of the points above.

Software and code

Policy information about availability of computer code

Data collection	Southern data was collected on a Typhoon FLA 9000 using the manufacturer's software and visualized using Image J 1.47v<br>EMSAs data collected on the Typhoon Trio+ scanner (Molecular Dynamics)<br>Crystallographic data were collected using software customized for each beamline. All software are publicly available with the respective instruments.
Data analysis	Bowtie(1.1.1) was used to align ChIP-seq reads and MACS(1.4.2) was used to call Orc1 peaks. DynaMO(v1.0) was used to analyze the spatial characteristics of H4K16ac and Orc1.<br>Crystallographic data were reduced by HKL2000 version V715. The structures were determined and refined by Phenix version dev-2689. Structures were built with Coot version 0.8.9.1.<br>EMSA data was quantified using ImageQuant software 5.2v (Molecular Dynamics). Apparent Kd determination was calculated with Prism 7 software (GraphPad Software).<br>The intact mass was deconvoluted using Intact Mass(Protein Metrics) and the MS/MS spectra were searched using Prosight Light software

For manuscripts utilizing custom algorithms or software that are central to the research but not yet described in published literature, software must be made available to editors/reviewers. We strongly encourage code deposition in a community repository (e.g. GitHub). See the Nature Research guidelines for submitting code & software for further information.

October 2018

Data

Policy information about availability of data

All manuscripts must include a data availability statement. This statement should provide the following information, where applicable:

- Accession codes, unique identifiers, or web links for publicly available datasets
- A list of figures that have associated raw data
- A description of any restrictions on data availability

All materials will be available by request. Coordinates of structures and structure factors will be deposited to the PDB bank, and PDB ID codes will be available prior to publication.

Field-specific reporting

Please select the one below that is the best fit for your research. If you are not sure, read the appropriate sections before making your selection.

☒ Life sciences ☐ Behavioural & social sciences ☐ Ecological, evolutionary & environmental sciences

For a reference copy of the document with all sections, see nature.com/documents/nr-reporting-summary-flat.pdf

Life sciences study design

All studies must disclose on these points even when the disclosure is negative.

Sample size	All Southern time points represent DNA extracts from samples containing hundreds of thousands of yeast cells. All samples were taken as consecutive time points from cultures at the same optical density. Sample size effects are therefore not expected.
Data exclusions	No data were excluded from the analysis
Replication	All Southern analyses were performed in at least 3 replicates with the exception of the A2P dosage series, which was performed twice. All analyses yielded consistent results. Only one representative Southern is shown for each experiment. All EMSAs were performed in at least 3 replicates, which was performed twice. All analyses yielded consistent results.
Randomization	Randomization was not relevant for Southern analysis as strains were isogenic. Thus no background effects are expected.
Blinding	Blinding was not relevant for Southern analysis as the raw data are shown.

Reporting for specific materials, systems and methods

We require information from authors about some types of materials, experimental systems and methods used in many studies. Here, indicate whether each material, system or method listed is relevant to your study. If you are not sure if a list item applies to your research, read the appropriate section before selecting a response.

Materials & experimental systems

n/a	Involved in the study	
☐	☒	Antibodies
☐	☒	Eukaryotic cell lines
☒	☐	Palaeontology
☒	☐	Animals and other organisms
☒	☐	Human research participants
☒	☐	Clinical data

Methods

n/a	Involved in the study	
☒	☐	ChIP-seq
☒	☐	Flow cytometry
☒	☐	MRI-based neuroimaging

Antibodies

Antibodies used	Anti-Histone H4 acetyl Lys16 (Active Motif, #39167) and anti-Histone H4 Abcam (ab7311).
Validation	Wilkins, B. J. et al. Genetically Encoding Lysine Modifications on Histone H4. ACS Chem. Biol. 10, 939–944 (2015). And, manufactures web sites.

Eukaryotic cell lines

Policy information about cell lines

Cell line source(s)	All Saccharomyces cerevisiae cell lines are isogenic and are of the SK1 background.

Authentication	Insertions of orc1 constructs were authenticated by Southern blotting to test for the presence and number of integrations. All strains subsequently underwent tetrad dissection and verification of genetic markers before diploids were constructed.
Mycoplasma contamination	n/a
Commonly misidentified lines (See ICLAC register)	n/a
